# Obesity Induces Disruption of Microvascular Endothelial Circadian Rhythm

**DOI:** 10.3389/fphys.2022.887559

**Published:** 2022-05-05

**Authors:** Caleb A. Padgett, Joshua T. Butcher, Steven B. Haigh, Andrew C. Speese, Zachary L. Corley, Cody L. Rosewater, Hunter G. Sellers, Sebastian Larion, James D. Mintz, David J. R. Fulton, David W. Stepp

**Affiliations:** ^1^ Vascular Biology Center, Augusta, GA, United States; ^2^ Department of Physiological Sciences, College of Veterinary Medicine, Oklahoma State University, Stillwater, OK, United States; ^3^ Division of Gastroenterology and Hepatology, Department of Medicine, College of Medicine, Medical University of South Carolina, Charleston, SC, United States; ^4^ Department of Pharmacology and Toxicology, Augusta, GA, United States; ^5^ Department of Physiology, Medical College of Georgia, Augusta University, Augusta, GA, United States

**Keywords:** circadian, obesity, vascular endothelium, physiology, vascular biology, endothelial NO synthase, NADPH oxidase

## Abstract

Obese individuals are at significantly elevated risk of developing cardiovascular disease (CVD). Additionally, obesity has been associated with disrupted circadian rhythm, manifesting in abnormal sleeping and feeding patterns. To date, the mechanisms linking obesity, circadian disruption, and CVD are incompletely understood, and insight into novel mechanistic pathways is desperately needed to improve therapeutic potential and decrease morbidity and mortality. The objective of this study was to investigate the roles of metabolic and circadian disruptions in obesity and assess their contributions in promoting vascular disease. Lean (*db/+*) and obese (*db/db*) mice were subjected to 12 weeks of constant darkness to differentiate diurnal and circadian rhythms, and were assessed for changes in metabolism, gene expression, and vascular function. Expression of endothelial nitric oxide synthase (eNOS), an essential enzyme for vascular health, was blunted in obesity and correlated with the oscillatory loss of the novel regulator cezanne (OTUD7B). Lean mice subjected to constant darkness displayed marked reduction in vasodilatory capacity, while endothelial dysfunction of obese mice was not further compounded by diurnal insult. Endothelial gene expression of essential circadian clock components was altered in obesity, but imperfectly phenocopied in lean mice housed in constant darkness, suggesting overlapping but separate mechanisms driving endothelial dysfunction in obesity and circadian disruption. Taken together, these data provide insight into the nature of endothelial circadian rhythm in obesity and suggest a distinct mechanism by which obesity causes a unique circadian defect in the vasculature.

## Introduction

Obesity is a leading risk factor for cardiovascular disease, affecting over 40% of Americans (Rooy and Pretorius, 2014; Powell-Wiley et al., 2021). A major co-morbidity of the obese state is disruption in activities that follow a circadian rhythm, resulting in disorders such as binge-eating, sleep apnea, and insomnia (Sinha et al., 1996; Froy, 2010; Garaulet et al., 2010). These disorders may exacerbate aberrant metabolism due to alterations in the timing of sleeping and eating patterns, imposing additional risk factors on an already strained physiology. Further supporting a link between metabolic disease and circadian disruption, otherwise healthy individuals exposed to circadian insults such as shift work or frequent regular jet lag exhibit aberrant control of insulin, glucose, triglycerides, and blood pressure (Martino and Sole, 2009; Torquati et al., 2018; Chellappa et al., 2019). Taken together, these findings suggest a role of the circadian clock in the development of cardiovascular disease in the context of metabolic disease (Tahira et al., 2011; Ribas-Latre and Eckel-Mahan, 2016).

We and others have demonstrated that alteration of either the central or peripheral circadian clock causes endothelial dysfunction in lean mice, a major vascular defect evident in obesity (Viswambharan et al., 2007; Anea et al., 2009; Nernpermpisooth et al., 2015). Central to proper endothelial function is a healthy balance between nitric oxide (NO) production and subsequent scavenging by reactive oxygen species (ROS), produced by eNOS and NOX1, respectively (Meza et al., 2019). In the obese vasculature, a clear and important role has emerged for aberrant signaling of NADPH oxidase 1 (NOX1) due to its ability to generate superoxide, a potent scavenger of NO, as we and others have demonstrated that NOX1 is the main source of ROS production in the obese vasculature (Thompson et al., 2017; Lassègue and Griendling, 2010). Mechanistically, this is driven by NOX1-derived superoxide reacting with available NO to form peroxynitrite, while simultaneously oxidizing the essential eNOS cofactor BH4, further lowering NO bioavailability and accelerating eNOS uncoupling, respectively (Das et al., 2014). Both eNOS and NOX1 have been reported to be under control of the circadian clock, making them attractive targets for discovering a link between circadian and vascular dysfunction (Shang et al., 2016; Early et al., 2018).

Currently, the mechanism by which obesity impacts endothelial cell circadian rhythm is incompletely understood, and therapeutic interventions to restore circadian rhythm and improve cardiovascular outcomes face a critical deficit of information (Tsimakouridze et al., 2015). The aim of this study was to determine how circadian dysrhythmia negatively impacts the endothelial cell phenotype in obesity, and to identify potentially novel targets to ameliorate obesity-induced circadian disruption and corresponding endothelial dysfunction with the goal of improving cardiovascular outcomes.

## Materials and Methods

### Animal Studies

All animal experiments were conducted under Institutional Animal Care and Use Committee (IACUC) approval and in accordance with the NIH Guide for the Care and Use of Laboratory Animals. Mice heterozygous for mutation in the leptin receptor (Jackson Labs; strain no. 000697) were crossed to generate lean (*db/+*) and obese (*db/db*) cohorts. PER2:Luciferase mice (Jackson Labs; strain no. 006852) were crossed with heterozygous (*db/+*) mice to generate lean and obese PER2:Luciferase reporter mice. Male mice were subjected to constant darkness (DD) beginning at 8 weeks of age for 12 continuous weeks and sacrificed immediately afterward at 20 weeks of age. Cohorts were sacrificed at four distinct time points: 6 a.m./ZT 0, 12 p.m./ZT 6, 6 p.m./ZT 12, and 12 a.m./ZT 18. Cohorts are labeled as lean or obese in the figures, with DD denoting cohorts housed in constant darkness. Constant darkness was employed in order to differentiate between diurnal and circadian rhythm-specific effects. Mice were anesthetized in an induction chamber with 4% isoflurane at 1 L/min O_2_ and decapitated.

### Blood Pressure Telemetry

Blood pressure telemetry was performed as described previously (Qiu et al., 2014). Briefly, mice were anesthetized in an induction chamber with 4% isoflurane at 1 L/min O_2_ and kept under sedation during implantation with DSI PAC10 transmitters by isolating the left carotid and threading the catheter from the carotid branch into the aortic arch. Batteries packs were then tunneled above the back right shoulder. Mice were allowed to recover for 7 days, then blood pressure recordings were initiated and continued for 7 days.

### Body Composition

Mice were subjected to whole-body nuclear magnetic resonance (NMR) in a Minispec Body Composition Analyzer (Bruker; Model no. LF90II) to determine body fat percentage. After sacrifice, organs were isolated and weights recorded (Supplementary Table S1).

### Metabolic Phenotype

Mice were housed in a Comprehensive Lab Animal Monitoring System (Columbus Instruments) to assess daily and nightly food and water intake, energy expenditure, respiratory rate, and activity. Mice were allowed to acclimate for 24 h, then monitored for 72 h for collection. Mice were fasted 4 h before sacrifice in order to perform metabolic testing. Blood samples were analyzed for fasting blood glucose using a standard glucometer (AlphaTrak) and for HbA1c using a multi-test A1CNow HbA1c system (PTS Diagnostics). Plasma was separated *via* centrifugation and analyzed for total cholesterol (FujiFilm), insulin (Alpco), free fatty acids (Sigma), and triacylglycerols (FujiFilm) (Supplementary Table S2).

### Endothelial Cell Isolation

Third and fourth order mesenteric arteries from euthanized mice were dissected, trimmed of visceral fat, and flushed with PBS. Vessels were minced and incubated in dispase/collagenase II digestion buffer at 37°C for 45 min. Homogenates were spun down, resuspended in PBS, and incubated with anti-CD31 microbeads (Miltenyi Biotec) at 4°C for 15 min. Cells were washed with PBS and applied to a magnetic column apparatus where 2 ml of flow through fraction was collected in PBS. The column was then removed from the magnet and flushed with 1 ml PBS to collect an endothelial fraction. Cells were spun down and resuspended in Trizol for RNA isolation.

### Gene Expression

RNA was isolated from endothelial fractions using Direct-Zol RNA MiniPrep Plus (Zymo). cDNA was synthesized using OneScript cDNA Synthesis SuperMix (ABM). qPCR was performed in a CFX-Connect Real-Time PCR Detection System (Bio-Rad) utilizing BrightGreen Express ×2 qPCR MasterMix - iCycler (ABM). Products were cycled at 95°C for 3 min followed by cycles of 95°C for 15s, 58.5°C for 15 s, and 72°C for 15s for 40 cycles. Expression fold change was calculated using the 2^−ΔΔCT^ method normalized to 18 s rRNA as an internal control. Briefly, average CT values for 18 s rRNA were subtracted from average CT values for the gene of interest to generate ΔCT. The average ΔCT value for control samples was subtracted from experimental ΔCT values to generate ΔΔCT. Expression fold change was calculated by 2^−ΔΔCT^. Data are presented as average expression fold change at four time points: 6 a.m., 12 p.m., 6 p.m., and 12 a.m., and double-plotted for continuous interpretation. Data were normalized to 6 a.m. samples. Sequences for all primers utilized are listed in Supplementary Table S3. For luciferase reporter studies, PER2 activity in 50 mg of pulverized aortic tissue was assessed using Renilla luciferase assay (Promega).

### Pressure Myography

Second and third order mesenteric arteries were dissected in ice-cold Krebs solution (118 mM NaCl, 25 mM NaHCO_3_, 11.1 mM d-glucose, 4.71 mM KCl, 2.56 mM CaCl_2_, 1.13 mM NaH_2_PO_4_, 7 mMMgCl_2_). Dissected arteries were trimmed of adipose tissue, cannulated and mounted on glass pipettes in 10 ml of Krebs solution at 37°C in a single vessel chamber (Living Systems Instrumentation). Vessels were pressurized to physiological pressure of 60 mmHg with a Pressure Servo Controller (LSI; Model no. PS-200-S) and heated to physiological temperature of 37°C with a Temperature Controller (LSI; Model no. TC-095). Vessels were allowed to reach equilibrium for 30 min, after which viability was assessed with 10 µL of saturated KCl. After 3 washes in 37°C Krebs solution, dose-response curves of acetylcholine, phenylephrine, and sodium nitroprusside (10^−9^—10^−3^ M) were generated, with 3 washes of Krebs solution between each curve. Microvessels were then washed 3 times in calcium-free Krebs solution and allowed to equilibrate for 45 min. Myogenic tone was assessed in 20 mmHg increments (20—120 mmHg) by recording inner and outer diameters.

### Statistical Analyses

Analyses between two groups were performed using student’s t-test, and analyses between multiple groups were performed using one-way ANOVA with Bonferroni post hoc correction. Analyses were performed using GraphPad Prism 9.1 (GraphPad Software Inc.). Data are expressed as mean ± standard error of the mean. A value of *p* < 0.05 was used as criteria for statistical significance.

## Results

### Obese Mice Display Marked Dysregulation of Cardiometabolic and Vascular Diurnal Rhythms

**FIGURE 1:**
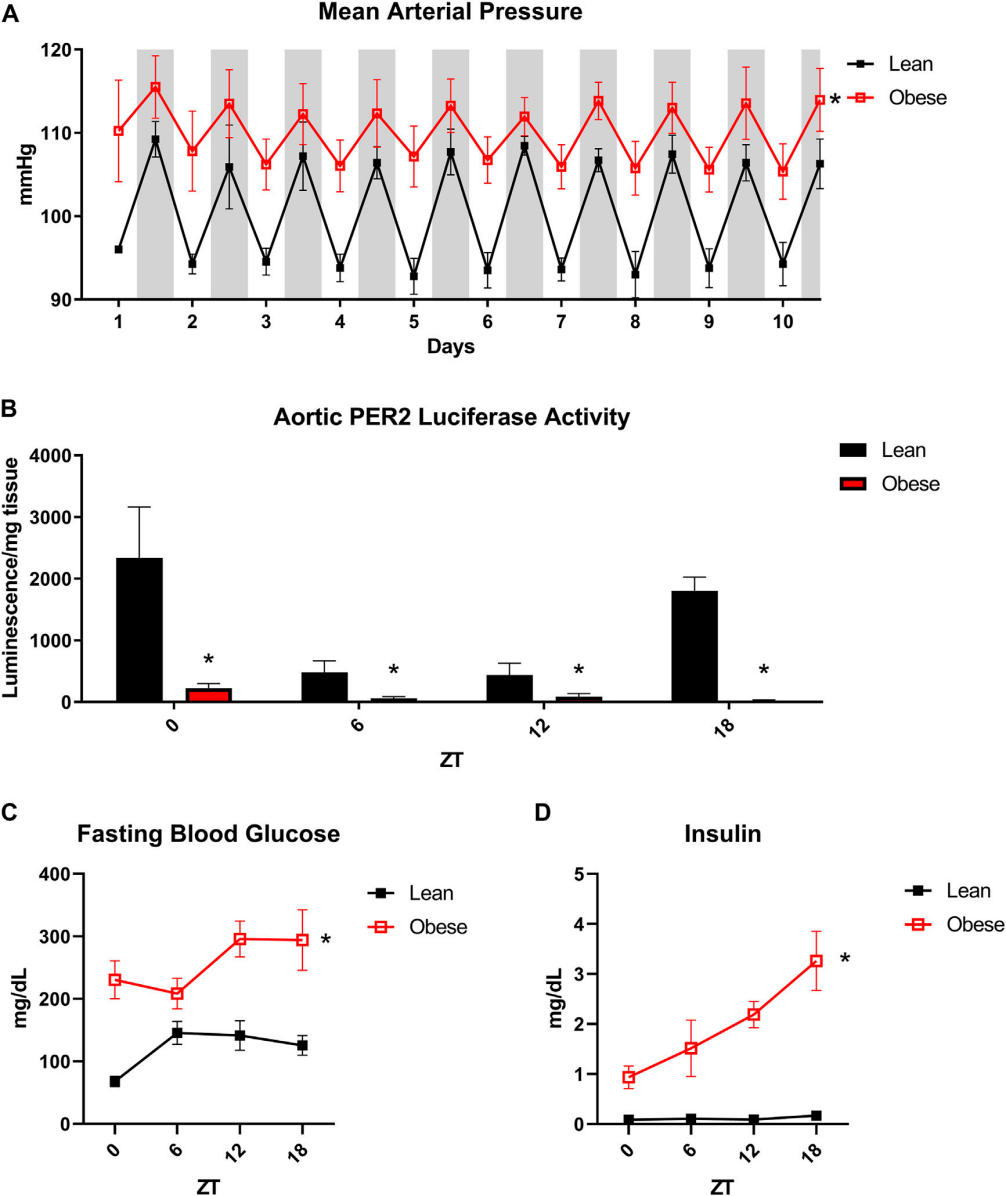
Obese *db/db* mice exhibit aberrant vascular and metabolic diurnal rhythms. **(A).** 24-h blood pressure telemetry from male 20-week old lean (*db/+*) and obese (*db/db*) mice (*n* = 3). **(B).** Luciferase activity from isolated aortas of lean and obese PER2:Luciferase reporter mice (*n* = 3). **(C).** Fasting blood glucose from male 20-week old lean (*db/+*) and obese (*db/db*) mice (*n* = 5). **(D).** Fasting plasma insulin from male 20-week old lean (*db/+*) and obese (*db/db*) mice (*n* = 8–10). All data are represented as mean ± SEM.

In order to assess rhythmic patterns in blood pressure, lean (*db/+*) and obese (*db/db*) mice were subjected to radiotelemetry. Obese mice displayed a significant increase in mean arterial pressure with a distinct non-dipping phenotype, indicative of obesity-induced hypertension (Figure 1A). Since obese mice displayed such a marked diurnal variation in blood pressure, we next investigated potential circadian abnormalities in the vasculature. In order to assess the oscillation of the peripheral clock in obesity, we generated lean and obese mice with a luciferase reporter for the core circadian clock component PER2. Obese mice exhibited marked blunting of PER2 oscillation in isolated aortae, indicating dysfunction of the clock in the vasculature (Figure 1B). Obese mice further exhibited circadian dysregulation of blood glucose (Figure 1C) and insulin levels (Figure 1D), further supporting the rationale of a unique convergence of cardiovascular, metabolic, and circadian disorders in the obese state. These observations demonstrate that the obese mouse model chosen for these studies recapitulate many of the aberrant circadian patterns of cardiometabolic function evident in human obesity.

### Obesity Causes Microvascular Endothelial Dysfunction and Loss of Circadian eNOS/NOX Balance

**FIGURE 2:**
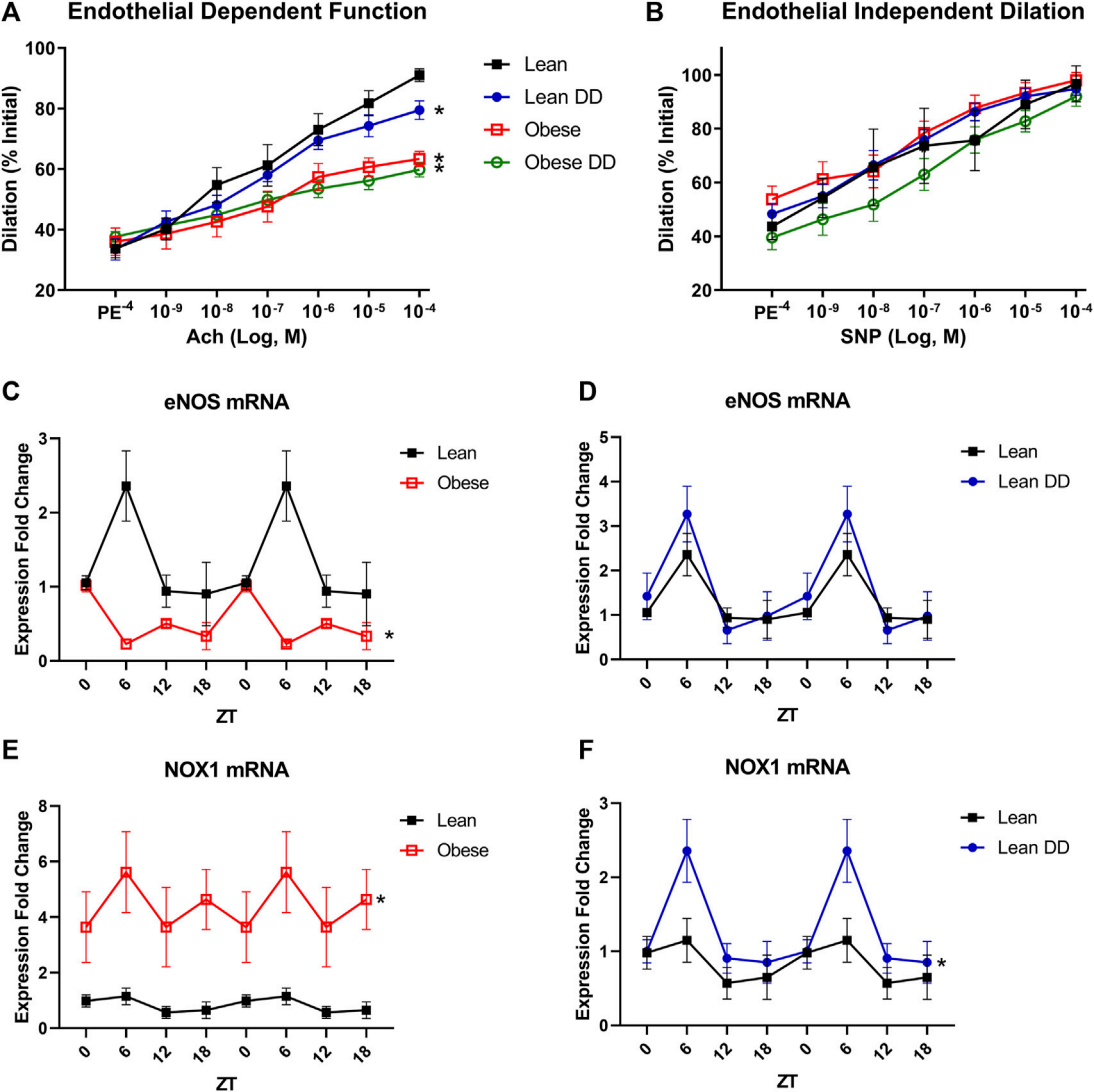
Obesity causes disruption of endothelial cell circadian rhythm and subsequent microvascular dysfunction. **(A).** Vasodilation (calculated as percent of initial resting diameter) to dose-response curve of acetylcholine in male 20-week old lean (*db/+*) and obese (*db/db*) mice with and without exposure to constant darkness (*n* = 4–6, one-way ANOVA). **(B).** Vasodilation (calculated as percent of initial resting diameter) to dose-response curve of sodium nitroprusside in male 20-week old lean (*db/+*) and obese (*db/db*) mice with and without exposure to constant darkness (*n* = 4–6, one-way ANOVA). **(C).** eNOS mRNA levels in freshly isolated mesenteric endothelial cells from male 20-week old lean (*db/+*) and obese (*db/db*) mice (*n* = 3–6). **(D).** eNOS mRNA levels in freshly isolated mesenteric endothelial cells from male 20-week old lean (*db/+*) mice with or without circadian disruption (*n* = 3–6). **(E).** NOX1 mRNA levels in freshly isolated mesenteric endothelial cells from male 20-week old lean (*db/+*) and obese (*db/db*) mice (*n* = 3–6). **(F).** NOX1 mRNA levels in freshly isolated mesenteric endothelial cells from male 20-week old lean (*db/+*) mice with or without circadian disruption (*n* = 3–6). All data are represented as mean ± SEM.

Since perturbations in circadian rhythm are so strongly associated with cardiovascular disease, we next assessed the microvascular function of lean and obese mice with and without diurnal disruption caused by 12-weeks housing in constant darkness. Obese mice displayed significantly impaired endothelium-dependent vasorelaxation of mesenteric arteries in response to a dose response curve of acetylcholine (Figure 2A). Disruption of diurnal rhythm caused endothelial dysfunction in lean mice, but did not worsen the already impaired endothelial response in obese mice. Neither diurnal disruption nor obesity affected alpha-adrenergic vasoconstriction in response to phenylephrine (Supplementary Figure S1A) or endothelium-independent vasodilation in response to sodium nitroprusside (Figure 2B), suggesting that the microvascular defect stems from loss of endothelial NO signaling. All changes in vascular function were independent of changes in baseline tone (Supplementary Figure S1B), maximum constriction (Supplementary Figure S1C), or resting internal diameter (Supplementary Figure S1D). In primary microvascular endothelial cells isolated from the mesenteric arteries of these mice, we discovered that circadian oscillation of eNOS expression was ablated and total expression decreased in obesity (Figure 2C), which was unaffected in lean mice with diurnal disruption (Figure 2D). Further, microvascular endothelial NOX1 expression was markedly increased in obesity (Figure 2E), and moderately upregulated at one time point in lean mice with diurnal disruption (Figure 2F). Thiobarbituric acid reactive substances (TBARS), an index of ROS overproduction, were likewise elevated in obese mice with and without exposure to constant darkness (Supplementary Figure S2). These data reveal that essential mediators of vasodilatory function display markedly disturbed circadian oscillation in obesity that are unique from the disruption seen in a model of constant darkness.

### Obesity Alters the Transcriptional Profile of the Endothelium

**FIGURE 3:**
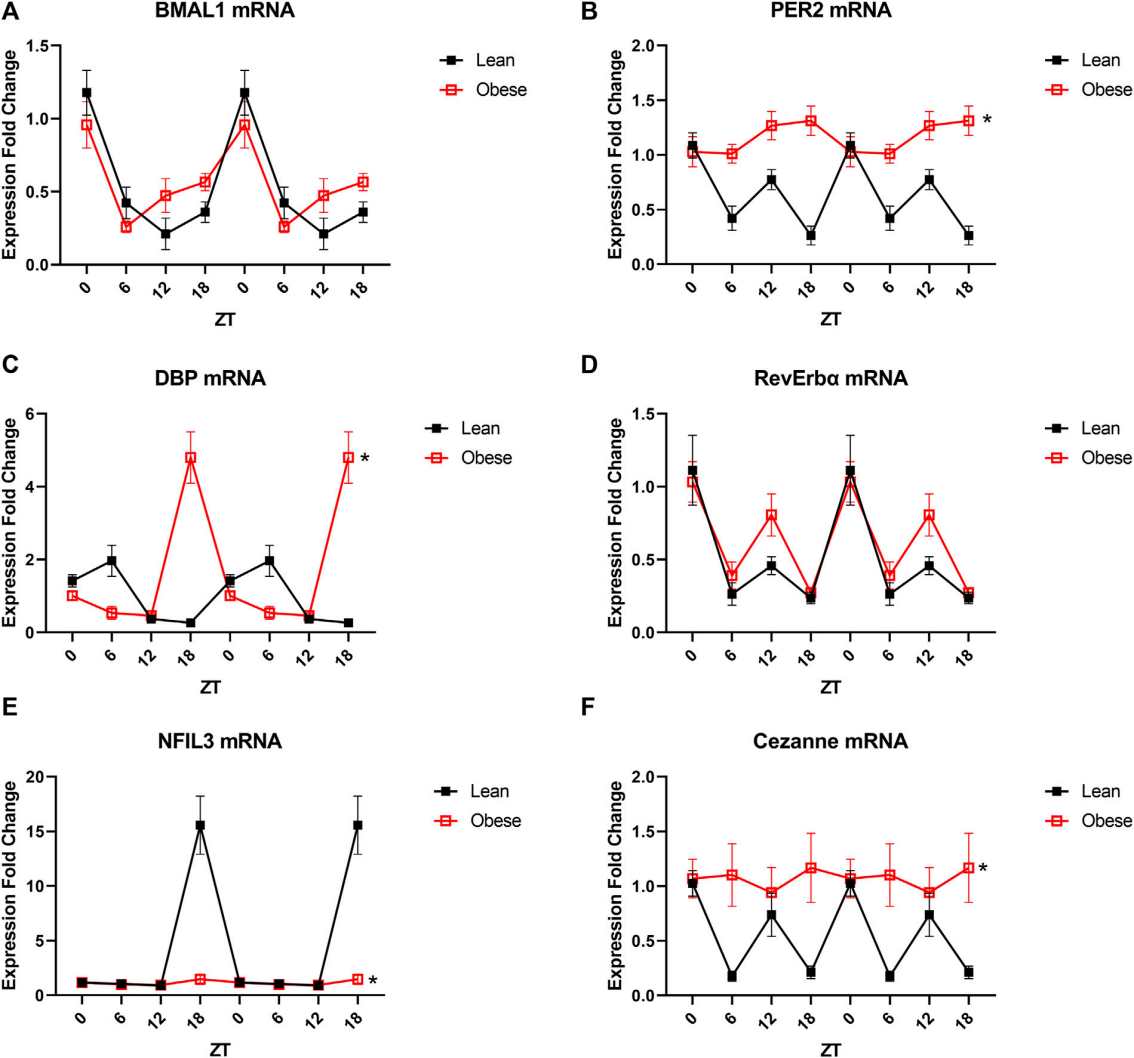
Obesity partially alters the transcriptional profile of endothelial cells. **(A).** BMAL1 mRNA levels in freshly isolated mesenteric endothelial cells from male 20-week old lean (*db/+*) and obese (*db/db*) mice (*n* = 3–6). **(B).** PER2 mRNA levels in freshly isolated mesenteric endothelial cells from male 20-week old lean (*db/+*) and obese (*db/db*) mice (*n* = 3–6). **(C).** DBP mRNA levels in freshly isolated mesenteric endothelial cells from male 20-week old lean (*db/+*) and obese (*db/db*) mice (*n* = 3–6). **(D).** RevErbα mRNA levels in freshly isolated mesenteric endothelial cells from male 20-week old lean (*db/+*) and obese mice (*db/db*) (*n* = 3–6). **(E).** NFIL3 mRNA levels in freshly isolated mesenteric endothelial cells from male 20-week old lean (*db/+*) and obese (*db/db*) mice (*n* = 3–6). **(F).** Cezanne mRNA levels in freshly isolated mesenteric endothelial cells from male 20-week old lean (*db/+*) and obese (*db/db*) mice (*n* = 3–6). All data are represented as mean ± SEM.

Since the only detectable microvascular defect was observed in the endothelium, we next assessed changes in microvascular endothelial circadian gene expression in obesity. Microvascular endothelial cells were selected for analysis since microvascular endothelial dysfunction is an early, independent predictor of cardiovascular disease and subsequent end-organ damage, especially in obesity. The core circadian clock component BMAL1 was unaltered in the obese endothelium (Figure 3A), while the core component PER2 exhibited a loss of oscillation across a 24-h time period (Figure 3B). Similarly, expression of the circadian output gene DBP was markedly altered in obesity (Figure 3C), while expression of the rhythmic integrator RevErbα exhibited only a modest change at one time point (Figure 3D). NFIL3, a circadian regulator frequently implicated in metabolic pathogenesis, was blunted in obesity (Figure 3E), as well as the novel eNOS regulator, Cezanne (Figure 3F). In order to further highlight the differences between diurnal and circadian disruption, data from all four cohorts of mice were plotted together for comparison (Supplementary Figures S3A–H). Taken together, these data demonstrate significant circadian impairment in obesity in multiple essential clock components as well as factors that directly influence endothelial and thereby vascular health.

### Diurnal Disruption Partially Alters the Transcriptional Profile of the Endothelium

**FIGURE 4:**
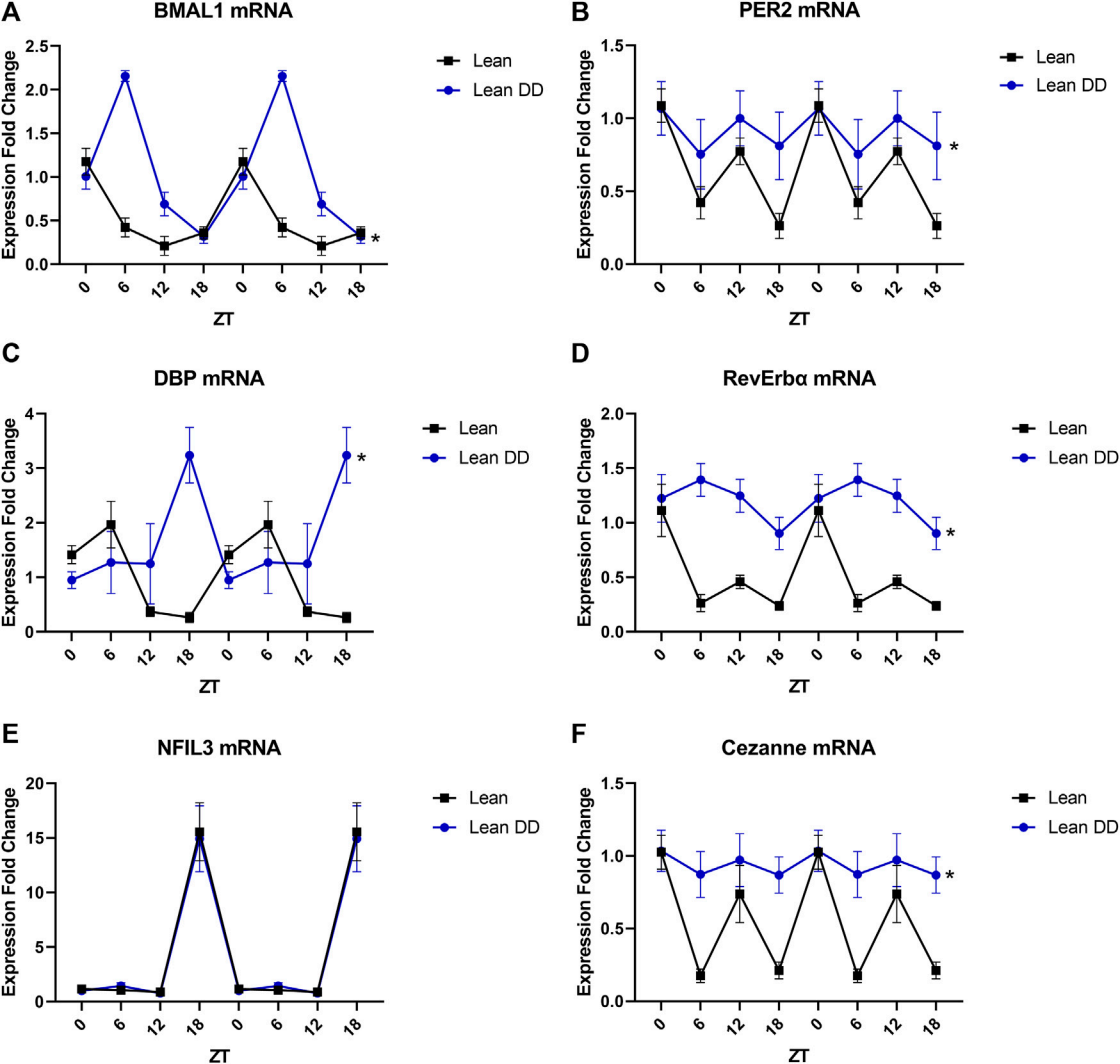
Diurnal disruption partially alters the transcriptional profile of endothelial cells. **(A).** BMAL1 mRNA levels in freshly isolated mesenteric endothelial cells from male 20-week old lean mice with and without exposure to constant darkness (*n* = 3–6). **(B).** PER2 mRNA levels in freshly isolated mesenteric endothelial cells from male 20-week old lean mice with and without exposure to constant darkness (*n* = 3–6). **(C).** DBP mRNA levels in freshly isolated mesenteric endothelial cells from male 20-week old lean mice with and without exposure to constant darkness (*n* = 3–6). **(D).** RevErbα mRNA levels in freshly isolated mesenteric endothelial cells from male 20-week old lean mice with and without exposure to constant darkness (*n* = 3–6). **(E).** NFIL3 mRNA levels in freshly isolated mesenteric endothelial cells from male 20-week old lean mice with and without exposure to constant darkness (*n* = 3–6). **(F).** Cezanne mRNA levels in freshly isolated mesenteric endothelial cells from male 20-week old lean mice with and without exposure to constant darkness (*n* = 3–6). All data are represented as mean ± SEM.

In order to interrogate the parallels between metabolic stress and circadian stress on endothelial clock function, lean mice with and without housing in constant darkness were subjected to the same analysis as performed in obese mice. Lean mice subjected to constant darkness exhibited differences in expression of the core components BMAL1 (Figure 4A) and PER2 (Figure 4B), with both alterations in periodicity and absolute level of expression. Corresponding shifts were further demonstrated in DBP (Figure 4C) and RevErbα (Figure 4D) expression. Interestingly, NFIL3 expression was unchanged by constant darkness (Figure 4E), while levels of Cezanne expression were blunted similarly to obese mice (Figure 4F). These data indicate that many circadian defects are unique to obesity and cannot be recapitulated in a model of constant darkness-induced circadian disruption.

## Discussion

Obesity induces circadian disruptions in behavior and physiology, a process that leaves a footprint on cellular gene expression that could contribute to both the genesis and progression of disease. While prior studies have described a variety of obesity-induced circadian disruptions, cell-specific changes in the circadian behavior of genes in endothelial cells have not been examined. In the current study, we provide the first detailed examination of clock components and the circadian behavior of genes central to the health of the endothelium in a validated model of obesity. We find that obesity profoundly depresses the vascular clock both *in situ* and specifically in endothelial cells and promotes the expression of pro-oxidant genotype known to be associated with impaired vascular function. Relevant considerations of these findings are the model, circadian impacts on endothelial function independent of obesity and the importance of the NOS/NOX axis in mediating endothelial health.

Impaired circadian regulation of blood pressure and blood glucose in obese mice recapitulate similar findings in obese humans (Kanbay et al., 2008; Poggiogalle et al., 2018; Moczulska et al., 2020). Frequently, patients with non-dipping hypertension exhibit worsened cardiovascular outcomes than those with dipping hypertension (Wolf et al., 2010). While the mechanism linking non-dipping hypertension and worsened outcomes is not completely understood, many postulate that increased blood pressure during sleep impairs its restorative nature, hampering the ability to adequately recover from the stresses of the day (Sayk et al., 2007; Noh, 2018). Likewise, obese patients exhibit poor control of nocturnal glucose, which ablates anticipatory metabolic responses to nutrition, causing dysregulation of insulin secretion, impairment of glucose storage, and further disruption of the circadian clock (Ribas-Latre and Eckel-Mahan, 2016; Chellappa et al., 2019). Our data in the *db/db* mouse model of obesity recapitulate these similar findings in humans, which further correlate with vascular circadian disruption. The knowledge of whether circadian disruption or obesity causes the other has proven to be elusive, but data across the literature agree that both pathologies amplify each other and converge on the presentation of cardiovascular disease (Froy, 2010; Shi et al., 2013).

Previous studies from our laboratory have identified oscillation patterns of essential circadian clock components in the whole vessels of obese mice, but not at the resolution of individual vascular cell components (Nernpermpisooth et al., 2015). Based on those initial results, we investigated how expression of select circadian genes oscillate differently in the endothelium of lean mice subjected to constant darkness, as well as in the endothelium of obese mice. Two essential findings arise from these data. First, that every essential component of the circadian clock need not be disrupted to cause or imply pathology. For example, our data indicate that the expression of the vital clock component BMAL1 is not altered in the endothelium of obese mice, which could indeed cast doubt on the degree of circadian disruption present in obesity. However, DBP, PER2, and NFIL3, and in turn many of the downstream genes they govern, all display marked dysregulation in obesity. Of particular interest is the absence of NFIL3 oscillation, which has been demonstrated to play an essential role in lipid handling and the progression of metabolic disease (Kang et al., 2017; Wang et al., 2017). While its role in the intestinal epithelium has been described, its role in the vascular endothelium is yet unknown, and presents an attractive potential target to better understand the role of the endothelial clock in anticipating and responding to dietary lipids. The second finding is that circadian disruption by constant darkness does not completely phenocopy the disruption seen in obesity. Healthy lean mice exposed to constant darkness displayed significant alterations in PER2 expression, but only modestly shifted the phase of BMAL1 and DBP. These data indicate that constant darkness reveals the core circadian clock mechanism, while obesity alters primarily downstream clock components and modulators. Due to the innate complexity of the clock mechanism, caution must be taken to validate models of circadian disruption before comparing them in other pathological states such as obesity and metabolic disease (Arble et al., 2010; Tsang et al., 2017).

We and others have consistently demonstrated the pathological effects of glucose on the endothelium in obesity, specifically in regards to imbalance of the eNOS/NOX1 axis (Belin de Chantemele and Stepp, 2012; Tsimakouridze et al., 2015; Zhu et al., 2015; Knapp et al., 2019). Data from this study confirm that both eNOS and NOX1 undergo oscillatory expression in the endothelium, and that in obesity, this axis shifts to favor an increase in NOX1-mediated ROS production and a pro-inflammatory phenotype. This imbalance favors decreased NO availability and corresponding impairment in endothelial vasodilatory capacity. Cezanne, or OTUD7B, is a novel deubiquitinase that regulates both NFκB and HIF1α expression, which in turn regulate NOX1 and eNOS expression, respectively (Enesa et al., 2008; Manea et al., 2010; Abe and Berk, 2013; Luong et al., 2013; Rodriguez-Miguelez et al., 2015). Cezanne has been demonstrated to undergo circadian oscillation and plays an important role in regulating immune response, however its role in the endothelium is not presently understood (Hou et al., 2017; Qi et al., 2020). Blunting of oscillatory Cezanne expression is hypothesized to dysregulate eNOS and NOX1, which correlates with our data. These data point to Cezanne as a potential molecular link between circadian disruption and endothelial dysfunction, and present an attractive target for future mechanistic studies.

In conclusion, data from our study demonstrate that obese mice display marked circadian dysregulation of blood pressure and blood glucose, both of which predict and precede acute cardiovascular events. We demonstrate that diurnal disruption causes endothelial dysfunction in healthy mice but does not compound endothelial dysfunction in obese mice. Data indicate that while rhythmic endothelial gene expression is indeed altered in obesity, it is not perfectly phenocopied in lean mice with diurnal disruption, providing important insight into the methods utilized to study peripheral circadian rhythm in mice with disturbed central rhythm. Taken together, these data identify a role of the circadian clock in regulating eNOS/NOX1 balance and thereby endothelial function and vascular health. The endothelial circadian clock presents an attractive therapeutic target to ameliorate vascular circadian disruption and corresponding cardiovascular disease.

## Data Availability

The raw data supporting the conclusions of this article will be made available by the authors, without undue reservation.

## Supplementary Material

The Supplementary Material for this article can be found online at: https://www.frontiersin.org/articles/10.3389/fphys.2022.887559/full#supplementary-material

Supplementary Figure S1 Microvascular function parameters. **(A)**. Vasoconstriction (calculated as percent of initial resting diameter) to dose-response curve of phenylephrine in male 20-week old lean *(db/+*) and obese (*db/db)* mice with and without exposure to constant darkness (*n*=4–6, one-way ANOVA). **(B)**. Percent tone (calculated as average resting diameter over maximum diameter) in male 20-week old lean *(db/+*) and obese (*db/db)* mice with and without exposure to constant darkness (*n*=4–6, one-way ANOVA). **(C)**. Vasoconstriction (calculated as percent of maximum diameter) to saturated potassium chloride in male 20-week old lean *(db/+*) and obese (*db/db)* mice with and without exposure to constant darkness (*n*=4–6, one-way ANOVA). **(D)**. Average microvessel resting diameter in male 20-week old lean *(db/+*) and obese (*db/db)* mice with and without exposure to constant darkness (*n*=4–6, one-way ANOVA). All data are represented as mean ± SEM.

Supplementary Figure S2 Plasma reactive oxygen species. Plasma levels of thiobarbituric acid reactive substances in male 20-week old lean *(db/+*) and obese (*db/db)* mice with and without exposure to constant darkness (*n*=5). All data are represented as mean ± SEM.

Supplementary Figure S3 Total qPCR of all mouse cohorts. **(A)**. eNOS mRNA levels in freshly isolated mesenteric endothelial cells from male 20-week old lean *(db/+*) and obese (*db/db)* mice with and without constant darkness (*n*=3–6). **(B)**. NOX1 mRNA levels in freshly isolated mesenteric endothelial cells from male 20-week old lean *(db/+*) and obese (*db/db)* mice with and without constant darkness (*n*=3–6). **(C)**. BMAL1 mRNA levels in freshly isolated mesenteric endothelial cells from male 20-week old lean *(db/+*) and obese (*db/db)* mice with and without constant darkness (*n*=3–6). **(D)**. PER2 mRNA levels in freshly isolated mesenteric endothelial cells from male 20-week old lean *(db/+*) and obese (*db/db)* mice with and without constant darkness (*n*=3–6). **(E)**. DBP mRNA levels in freshly isolated mesenteric endothelial cells from male 20-week old lean *(db/+*) and obese (*db/db)* mice with and without constant darkness (*n*=3–6). **(F)**. RevErbα mRNA levels in freshly isolated mesenteric endothelial cells from male 20-week old lean *(db/+*) and obese (*db/db)* mice with and without constant darkness (*n*=3–6). **(G)**. NFIL3 mRNA levels in freshly isolated mesenteric endothelial cells from male 20-week old lean *(db/+*) and obese (*db/db)* mice with and without constant darkness (*n*=3–6). **(H)**. Cezanne mRNA levels in freshly isolated mesenteric endothelial cells from male 20-week old lean *(db/+*) and obese (*db/db)* mice with and without constant darkness (*n*=3–6). All data are represented as mean ± SEM.

## Abbreviations

Ach, acetylcholine; BMAL1, brain and muscle ARNT-like 1; CVD, cardiovascular disease; DBP, d site of albumin promoter binding protein; DD, dark/dark cycle; eNOS, Endothelial Nitric Oxide Synthase; NFIL3, nuclear factor, interleukin 3 regulated; NO, nitric oxide; NOX1, NADPH oxidase I; PE, phenylephrine; PER2, period circadian protein homolog 2; RevErbα, nuclear receptor subfamily 1 group D member 1; ROS, reactive oxygen species; SNP, sodium nitroprusside; TBARS, thiobarbituric acid reactive substances.
