# Supplementary figures and images for: Obesity Induces Disruption of Microvascular Endothelial Circadian Rhythm
Source: Front Physiol. 2022 May 5;13:887559. doi: 10.3389/fphys.2022.887559 (PMC9119407; doi:10.3389/fphys.2022.887559)

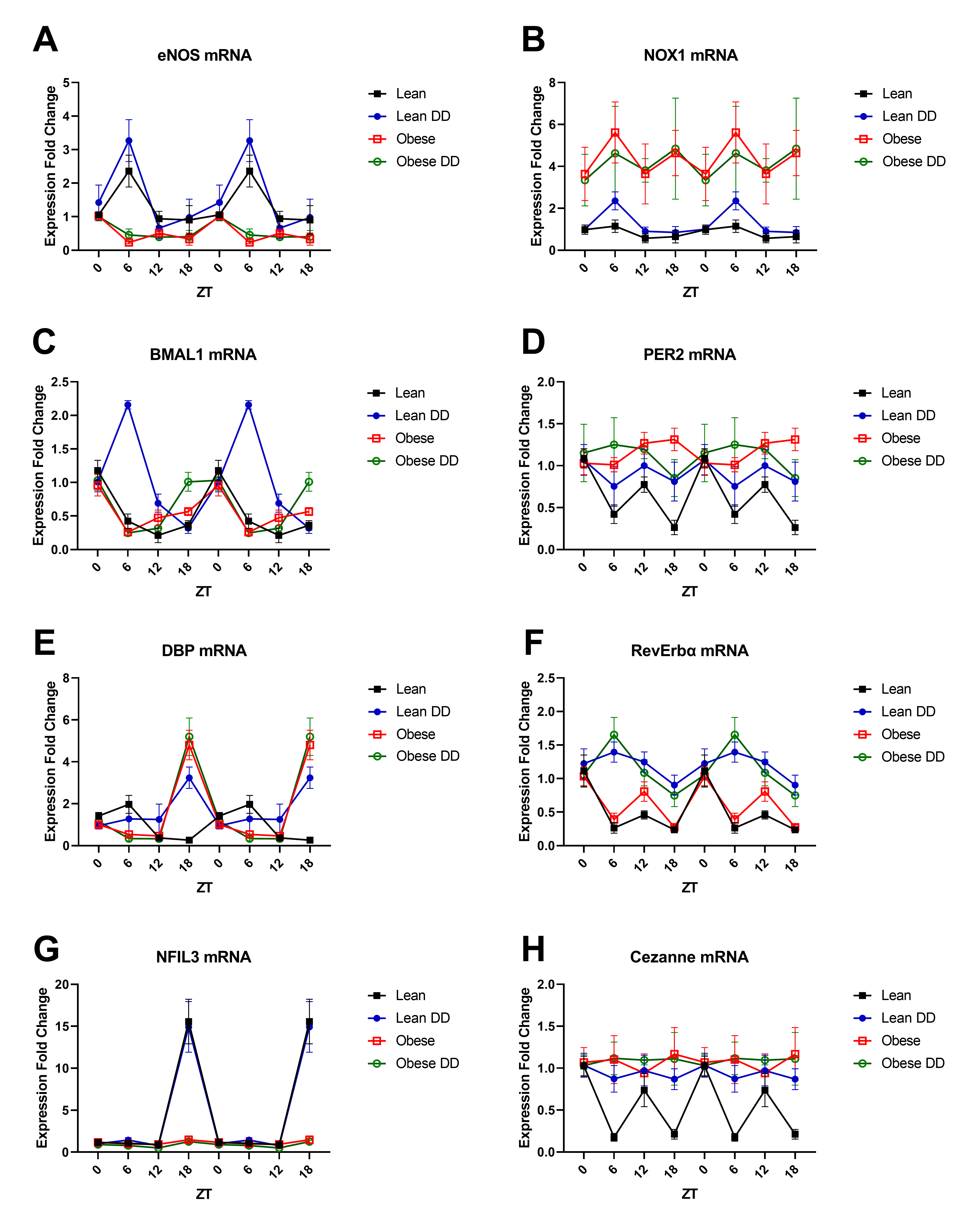

Supplement: Supplementary file 1 [file Image3.TIF]

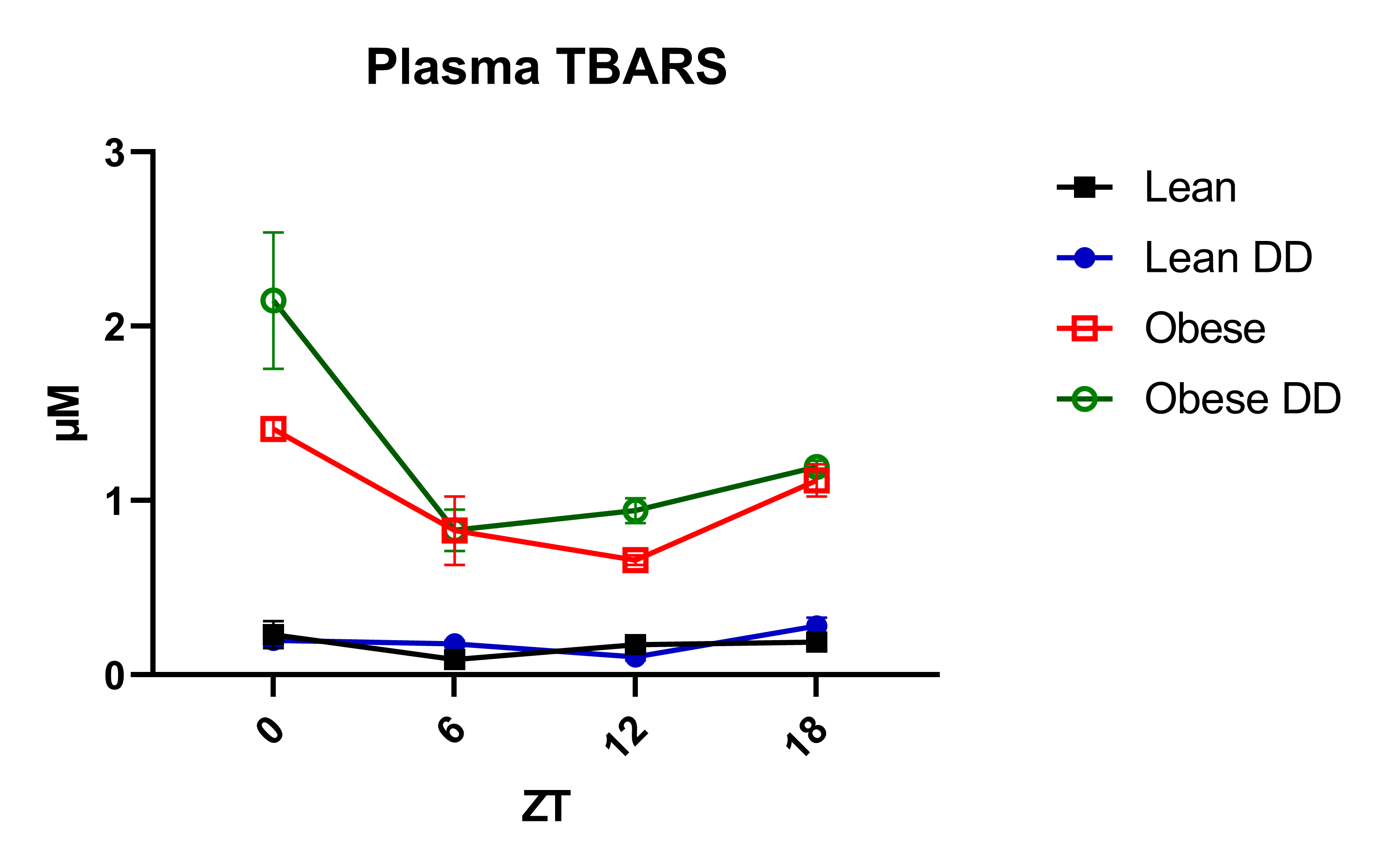

Supplement: Supplementary file 2 [file Image2.TIF]

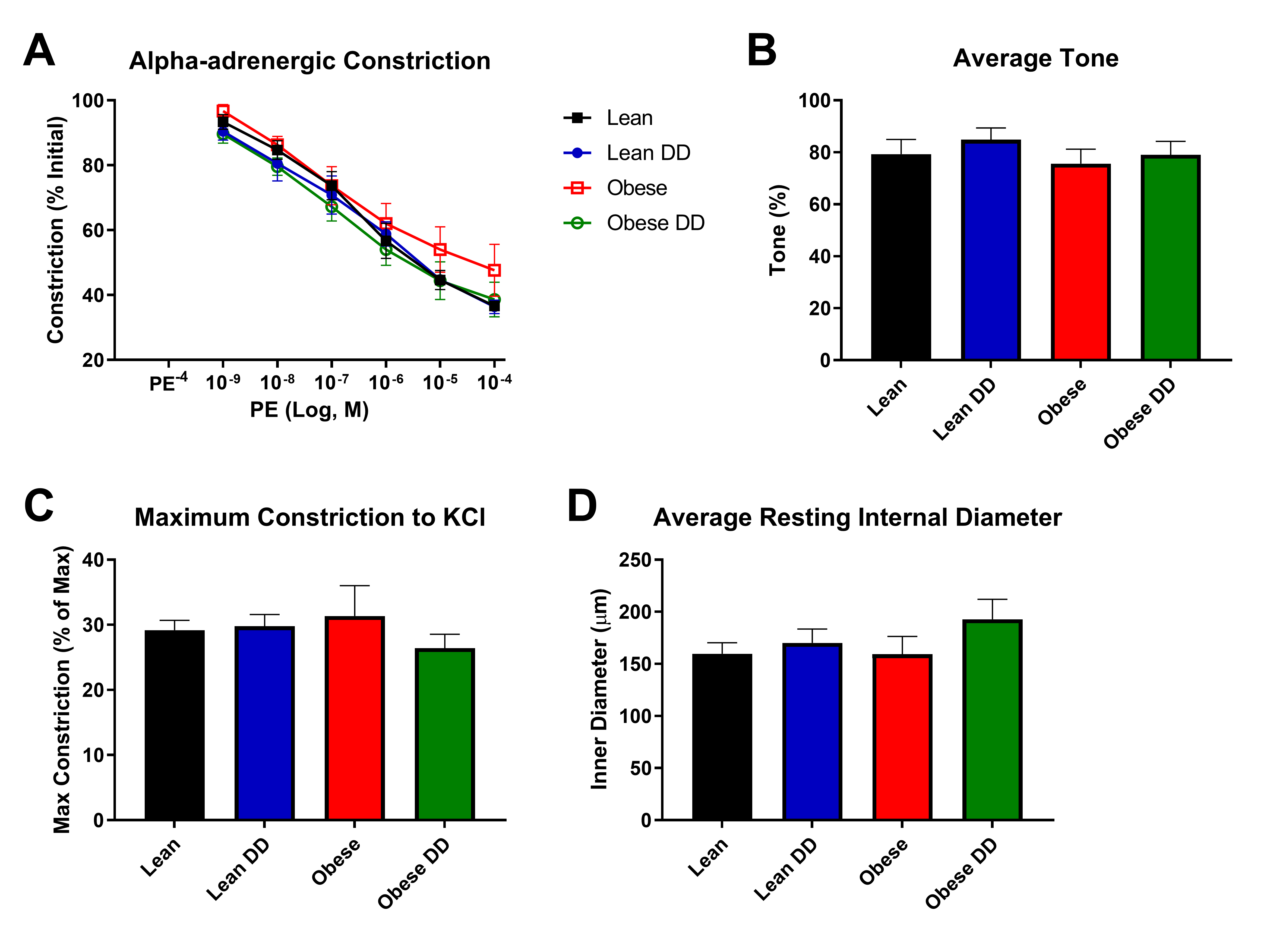

Supplement: Supplementary file 3 [file Image1.TIF]
